# Is Dupilumab as Effective in Intrinsic Atopic Dermatitis as It Is in Extrinsic Atopic Dermatitis?

**DOI:** 10.3390/jcm12062189

**Published:** 2023-03-11

**Authors:** Federica Gelato, Luca Mastorino, Ekaterina Stepkina, Giovanni Cavaliere, Simone Ribero, Pietro Quaglino, Michela Ortoncelli

**Affiliations:** Dermatology Clinic, Department of Medical Sciences, University of Turin, 10126 Turin, Italy

**Keywords:** atopic dermatitis, dupilumab, intrinsic atopic dermatitis

## Abstract

Atopic dermatitis (AD) can be subclassified into the more frequent extrinsic type (EAD), with elevated serum IgE levels and frequent association with other atopic conditions, and the less frequent intrinsic type (IAD), with normal IgE levels and no history of atopy. This retrospective study has the objective to compare the efficacy of dupilumab therapy in patients with IAD versus EAD in a real-life setting. We studied a group of 360 patients treated with dupilumab for moderate-to-severe AD of whom 49 had IAD (IgE < 200 kU/L and no history of other atopic conditions) and 311 had EAD (IgE ≥ 200 kU/L and/or history of atopy). There were no statistically significant differences in the achievement of EASI75 between IAD and EAD patients either at 16, 32, or 48 weeks (61% vs. 50%; 66% vs. 60%; and 53% vs. 65%, respectively). Similarly, there were no statistically significant differences in the achievement of EASI90 or the reduction in NRSpp, NRSsd, and DLQI at each timepoint. Additionally, mean absolute eosinophils and IgE values were significantly higher in the EAD group at all timepoints. This study confirms that dupilumab, targeting the Th2 pathway, which is known to be overexpressed in all AD phenotypes, appears to be equally effective in the two populations regardless of IgE levels.

## 1. Introduction

Atopic dermatitis (AD) is a common inflammatory disease of the skin characterized by intense pruritus and chronic or relapsing eczematous lesions. The Global Burden of Disease study showed a prevalence of 15–20% among children and up to 10% among adults with a notable impact on the quality of life of patients and their families and with significant economic repercussions [1].

AD is frequently associated with a personal or family history of atopic conditions, such as bronchial asthma and allergic rhinitis, which is why the term “atopic dermatitis” was chosen [2,3].

In 2003, the Nomenclature Review Committee of the World Allergy Organization proposed a new nomenclature for allergies in which the term “atopy’’ refers to a genetic predisposition to become immunoglobulin E (IgE) sensitized to allergens commonly found in the environment to which everyone is exposed but to which most people do not produce a long-term IgE antibody response [4].

Most patients diagnosed with AD have high concentrations of total IgE, positive allergen-specific IgE levels, and positive skin prick tests with aeroallergens and/or food allergens. However, there are patients with similar clinical features of AD but without detectable sensitization to inhalant or food allergens [5]. For this reason, among patients with a clinical diagnosis of AD, a distinction into two subgroups has been proposed. Similar to the extrinsic and intrinsic types of asthma, the term “intrinsic AD” (IAD) and “extrinsic AD” (EAD) have been suggested. For IAD, other similar terms such as “non-allergic AD” or “non-atopic eczema” have been introduced to describe AD patients without allergen-specific IgE [4,6]. Since these patients have no demonstrable atopy, the term “atopiform dermatitis” has also been coined to avoid the link with true atopy [7].

Since total serum IgE values are correlated with the allergen-specific IgE status, total IgE can be regarded as a clinically useful parameter to expectedly differentiate between the extrinsic type, with increased IgE levels, and the intrinsic type, with normal IgE values, in both adults and children [5,8].

The prevalence of IAD has been reported between 15% and 45% in different studies, and it is characterized by a female predominance, a later age at onset, and a more frequent distribution in the head and neck area (H&N) [5,9]. Instead, no histological differences were observed in the two groups. The typical findings are similar in both types of AD: epidermal spongiosis and dermal lymphocytic infiltrate in the acute-phase lesions and acanthosis in the chronic phase. Activated T cells predominate the dermal cell infiltrate, but other cells such as mast cells, eosinophils, and antigen-presenting dendritic cells are also present. In IAD and EAD, different cytokine patterns of involved T cells have been observed in peripheral blood, as well as in lesional skin biopsies. EAD patients have been found to express elevated levels of interleukin (IL)-4 and IL-13 with an increased production of IgE compared to patients with IAD [5]. In addition, patients with IAD were found to have a greater increase in the Th1 signal and more pronounced Th17/Th22 activation [10].

Taking this different cytokine production into account, we wondered whether dupilumab, an inhibitor of both IL-4 and IL-13 signaling, has less efficacy in patients with IAD.

In the literature, very few studies have evaluated the efficacy of dupilumab by stratifying patients according to normal versus increased IgE values, following a “one-size-fits-all” treatment approach [11,12]. With this study, we want to enrich the literature by comparing the efficacy of dupilumab in patients with IAD versus EAD in a real-life setting.

## 2. Materials and Methods

### 2.1. Study Population

We performed a retrospective observational study with the aim of comparing the efficacy of dupilumab therapy in patients with EAD versus IAD in a real-life setting.

Patients aged ≥12 years with moderate-to-severe AD who started treatment with dupilumab at the Dermatology Clinic of the Turin University Hospital between January 2019 and March 2022 were included in this study.

AD was diagnosed according to the Hanifin and Rajka criteria [13], and IAD was defined by total serum IgE levels < 200 kU/L and the absence of personal or family history of other atopic conditions such as bronchial asthma and allergic rhino-conjunctivitis according to Schmid (-Grendelmeier) et al. [5]. Adult patients had to have an EASI (eczema area severity index) ≥ 24, while adolescents required an EASI ≥ 24 or one of the following criteria: localization in sensitive or visible areas, an NRSpp (numerical rating scale peak of pruritus) ≥ 7, or a CDLQI (children’s dermatology life quality index) ≥ 10.

All adult patients received an initial dose of 600 mg dupilumab and subsequently 300 mg every other week administered as subcutaneous injection. Adolescents (aged 12–17), if they weighed less than 60 kg, received an initial dose of 400 mg and then 200 mg every other week, whereas if they weighed more than 60 kg, the same dosage was used as for adults. Patients were examined before starting dubilumab therapy (T0) and were reevaluated at 16 (T1), 32 (T2), and 48 (T3) weeks. At T0, demographic characteristics, such as age, sex, BMI, age of onset, family history of atopy, predisposition to allergic conjunctivitis and recurrent herpetic recurrences, and history of parasitic infections, were collected. At each visit, blood count, leukocyte formula (with particular attention to eosinophils), IgE, and LDH were assessed; patients completed the DLQI (dermatology life quality index) or the CDLQI and a POEM (patient-oriented eczema measure) before each visit. Disease severity was assessed by EASI, with special attention to H&N EASI; pruritus was assessed as NRSpp and NRSsd (sleep disturbance). The occurrence of adverse events (EAs) was also monitored at each visit.

### 2.2. Objectives

The aim of this study was the comparison of the efficacy of dupilumab therapy in patients with IAD versus EAD. These two groups were evaluated on the basis of improvements in EASI scores and H&N EASI at each T, the proportion of patients achieving EASI75 and EASI90 at each T, the percentage change from the baseline in worst pruritus NRS and NRSsd at each T, and improving quality of life by comparing DLQI and POEM scores at each T. LDH and eosinophil values were also compared in the two groups at each T.

### 2.3. Statistical Analysis

Quantitative variables were described by means with standard deviations (SD), while qualitative variables by percentages and absolute values.

Specifically, statistical analysis was performed using the Mann–Whitney U-test to compare independent variables with non-normal distributions.

For categorical variables, the Chi-square test was used, applying linear regression with Pearson’s index for correlations. Statistical significance was considered as a *p*-value < 0.05.

All analyses were performed with STATA software version 10 (STATA Corporation, College Station, TX, USA).

## 3. Results

At the baseline, 360 patients were included in this study, of which 49 (14%) patients had IAD and 311 (86%) had EAD. Among the IAD patients, 25 (52%) were female and 24 (49%) were male, while the among the EAD patients, 133 (43%) and 178 (57%), respectively.

As shown in Table 1, in the group of patients with IAD, the mean EASI at baseline was 18.2 (SD ± 11.97) and 24.04 (SD ± 10.34) in the EAD group (*p*-value < 0.01); furthermore, in the IAD group, there was an H&N EASI of 2.6 (SD ± 2.09), while in the EAD group, it was 3.33 (SD ± 2.1) (*p*-value < 0.05). At T0, patients with EAD had significantly higher mean IgE values than those with IAD (3949.74 ± 5208.77 vs. 70.64 ± 60.05, *p* < 001), as expected according to the definition of the two groups. At the baseline, there was also no statistically significant difference between the mean DLQI of the IAD group (14.13 ± 7.56) and the mean DLQI of the EAD group (14.88 ± 7.07) (*p*-value 0.496). There were also no differences in the POEM; the mean value reported by IAD patients was 19.19 ± 6.56, while in the EAD group, it was 20.8 ± 6.31 (*p*-value 0.094).

Among patients included in this study, 38 IAD patients and 234 EAD patients reached T1, at which time the mean EASI was 2.78 (SD ± 5.62) in the IAD group and 3.88 (SD ± 5.13) in the EAD group (*p*-value 0.028). At T2, 35 IAD patients had a mean EASI of 1.79 (SD ± 2.97), and 212 EAD patients had a mean EASI of 2.96 (SD ± 3.78) (*p*-value 0.011). At T3, 30 IAD patients had a mean EASI of 1.61 (SD ± 1.6), and 160 EAD patients had a mean EASI of 2.75 (SD ± 4.04) (*p*-value 0.222).

Thus, patients with IAD showed lower mean EASI values at T0, T1, and T2 than patients with EAD, whereas at T3, this difference nullifies.

There were no statistically significant differences between the two groups in the achievement of EASI75 at either T1, T2, or T3. The percentage of patients achieving EASI75 in patients with IAD versus patients with EAD was 61% vs. 50% (*p*-value 0.248) at T1, 66% vs. 60% (*p*-value 0.514) at T2, and 53% vs. 65% (*p*-value 0.224) at T3.

Similarly, there was no statistically significant difference in the achievement of EASI90 in patients with IAD and AED: 42% vs. 35% (*p*-value 0.352) at T1, 57% vs. 40% (*p*-value 0.059) at T2, and 40% vs. 47% (*p*-value 0.488) at T3.

**Figure 1:**
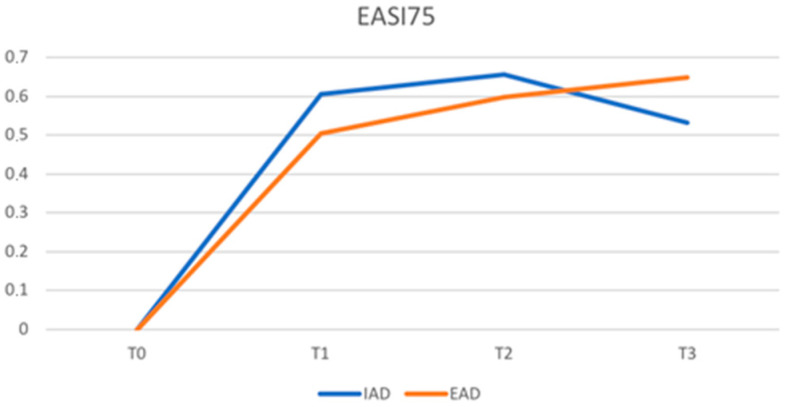
Proportion of patients achieving a 75% improvement in the eczema area and severity index (EASI75) (IAD = intrinsic atopic dermatitis; EAD = extrinsic atopic dermatitis).

**Figure 2:**
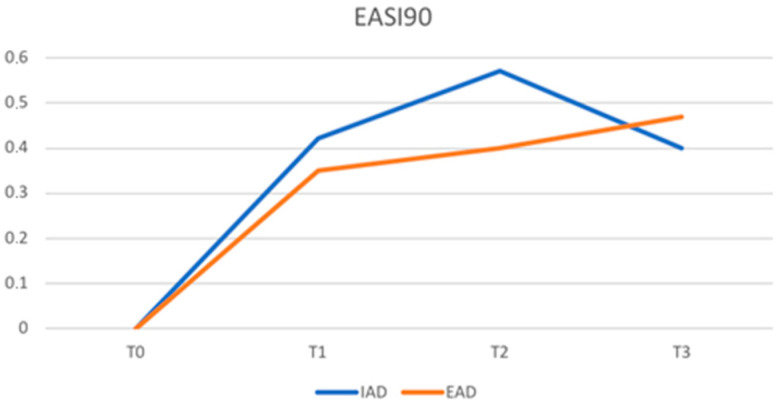
Proportion of patients achieving a 90% improvement in the EASI (EASI90) (IAD = intrinsic atopic dermatitis; EAD = extrinsic atopic dermatitis).

Figure 1 and Figure 2 show, respectively, the trend of EASI75 and EASI90 at various timepoints. Patients with IAD show a faster response up to T2 and then reverse the trend compared to EAD.

The mean H&N EASI in patients with IAD compared to those with EAD was 0.54 (SD ± 0.99) vs. 0.98 (SD ± 1.28) (*p*-value 0.17) at T1, 0.39 (SD ± 0.68) vs. 0.76 (SD ± 1.12) (*p*-value 0.46) at T2, and 0.38 (SD ± 0.62) vs. 0.73 (SD ± 1.02) (*p*-value 0.78) at T3.

With regard to itch scores, the mean NRSpp in IAD patients was 2.74 (SD ± 2.58) and 3.02 (SD ± 3.02) in EAD patients at T1 (*p*-value 0.50), 2.3 (SD ± 2.51) vs. 2.84 (SD ± 2.27) at T2 (*p*-value 0.112), and 2.54 (SD ± 2.10) vs. 2.66 (SD ± 2.33) at T3 (*p*-value 0.998).

**Figure 3:**
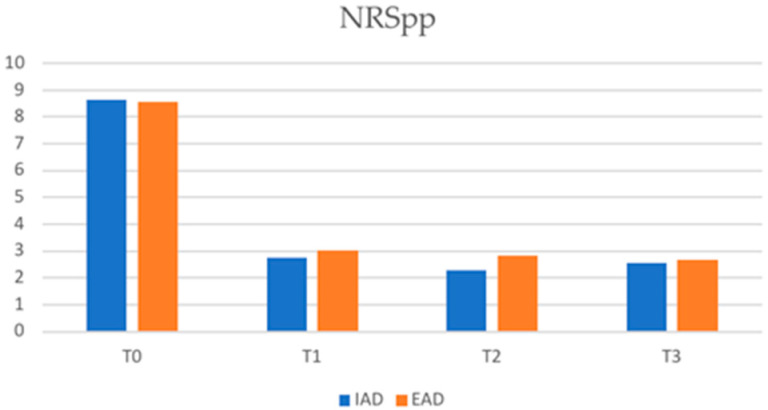
Numerical rating scale pick pruritus (NRSpp) in IAD and EAD patients (IAD = intrinsic atopic dermatitis; EAD = extrinsic atopic dermatitis).

**Figure 4:**
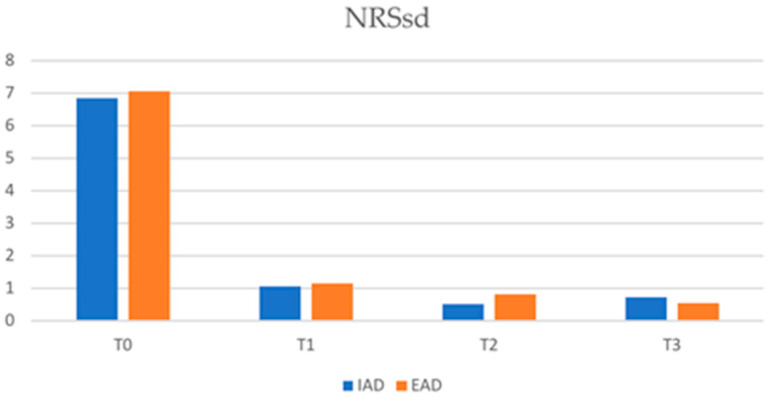
Numerical rating scale sleep disturbance (NRSsd) in IAD and EAD patients (IAD = intrinsic atopic dermatitis; EAD = extrinsic atopic dermatitis).

At T1, the mean NRSsd in IAD patients was 1.06 (SD ± 2.03) and 1.15 (SD ± 2.2) in EAD patients (*p*-value 0.759). The mean NRSsd in IAD vs. EAD patients was 2.3 (SD ± 2.51) vs. 2.84 (SD ± 1.68) at T2 (*p*-value 0.781) and was 0.75 (SD ± 2.15) vs. 0.55 (SD ± 1.68) at T3 (*p*-value 0.781). The reduction trend in NRSpp and NRSsd in the two study groups is shown in Figure 3 and Figure 4, respectively.

As for quality of life, the DLQI score was 4.79 ± 5.19 in the IAD group vs. 4.44 ± 5.01 in the EAD group at T1 (*p*-value 0.522), 3.88 ± 4.75 vs. 4.00 ± 4.99 at T2 (*p*-value 0.663) and 3.14 ± 2.86 vs. 3.24 ± 3.98 at T3 (*p*-value 0.679).

**Figure 5:**
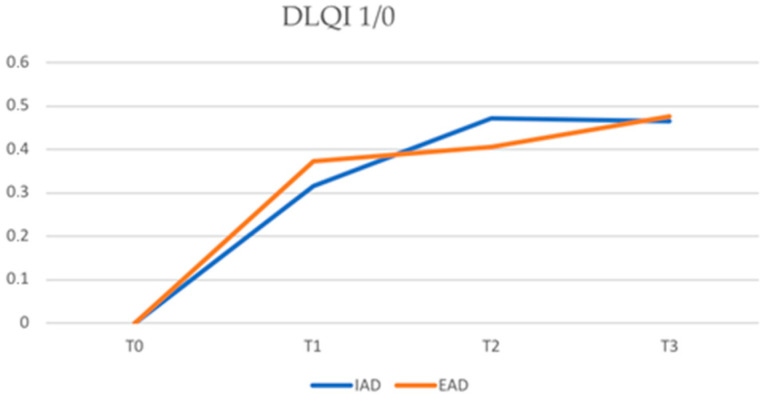
Proportion of patients achieving a DLQI of 1/0 in IAD and EAD. DLQI = dermatology life quality index (IAD = intrinsic atopic dermatitis; EAD = extrinsic atopic dermatitis).

The percentage of patients with EAD who achieved a DLQI of 1/0 compared to those with IAD was: 75.24% vs. 77.55% at T1, 64.31% vs. 69.39% at T2, and 48.55% vs. 57.14% at T3 (Figure 5). No statistically significant differences were found in the achievement of a DLQI of 1/0 in the two study groups (*p*-value > 0.05).

Mean IgE values were significantly higher, although decreasing over time, in the EAD group than in the IAD group at all timepoints (2159.10 ± 3243.13 vs. 44.81 ± 40.30 at T1, 1348.92 ± 1816.90 vs. 38.85 ± 39.39 at T2, and 997.23 ± 1376.00 vs. 23.69 ± 16.72 at T3; *p* < 0.001).

**Figure 6:**
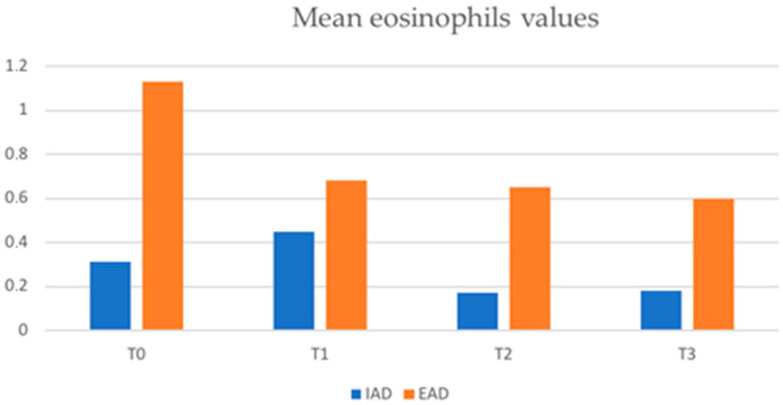
Mean eosinophils values (×10^9^/L) in IAD and EAD patients (IAD = intrinsic atopic dermatitis; EAD = extrinsic atopic dermatitis).

The mean absolute values of eosinophils in the two groups were also compared and were statistically higher in patients with EAD than in those with IAD at all timepoints (Figure 6). Values were 1.13 ± 9.07 × 10^9^/L vs. 0.31 ± 0.39 × 10^9^/L at T0 (*p* < 0.001), 0.68 ± 0.75 × 10^9^/L vs. 0.45 ± 1.24 × 10^9^/L at T1 (*p* < 0.001), and 0.65 ± 1.05 vs. 0.17 ± 0.11 × 10^9^/L at T2 (*p* < 0.001).

With regard to mean LDH values, no statistically significant differences were found in the two groups at any timepoint.

## 4. Discussion

We evaluated the possible difference between the H&N EASI in the two groups as this parameter appears to have an important impact in measuring quality of life compared to other body area EASIs [14]. In contrast to what has previously been shown by other studies [5,9], our patients with EAD had a higher mean H&N EASI than patients with IAD at the baseline. During treatment with dupilumab, these values remained higher in the EAD group, although without a statistically significant difference and with an irregular trend, probably due to red face events.

The mean EASI values were significantly higher in patients with EAD at T0 as well as at T1 and T2; only at T3, after 48 weeks of treatment, was this difference no longer significant. Therefore, patients with EAD in our sample show a higher disease burden at the baseline that remains higher than patients with IAD in the first 32 weeks of dupilumab treatment. Nevertheless, the percentage of patients achieving an EASI75 is high in both the IAD and EAD groups (61% vs. 50% at T1, 66% vs. 60% at T2, and 53% vs. 65% at T3), and no statistically significant differences were found in the two groups. Patients with IAD showed a faster response up to T2 and then reversed the trend compared to EAD. Similarly, patients in the two groups achieved an EASI90 in comparable percentages, demonstrating equal efficacy of dupiluamb in patients with IAD and EAD.

It is well known that AD is a Th2-type inflammatory disease. However, patients with EAD present high levels of type 2 cytokines, including IL-4, IL-5, and IL-13, with an increased eosinophil count, whereas the levels of these cytokines are relatively lower, although increased compared to normal, in IAD. The latter is also characterized by increased Th1 signaling and more pronounced Th17/Th22 activation with an overproduction of IFN-g that may further downregulate IgE production [10].

These mechanisms may explain the higher, since baseline, serum eosinophil levels and the mean EASI values in EAD compared to IAD obtained in our study.

In our study, no statistically significant differences between the two groups of patients were recorded regarding the assessment of quality of life, both in terms of the reduction in DLQI and the achievement of a DLQI of 1/0 or in the reduction in NRSpp and NRSsd. This shows that dupilumab is responsible for an improvement in the patients’ quality of life and a reduction in itching in both AD subtypes.

Since increased Th2 levels are a common feature across the AD spectrum, targeting this axis should theoretically be beneficial for all AD phenotypes [9].

This study confirms that dupilumab, by reducing IL-4 and IL-13 signaling, Th2-associated interleukins, appears to be equally as effective in the two study populations regardless of IgE levels, as previously shown by Hamilton et al. [11].

It can also be seen that the efficacy of dupilumab is not closely related to the reduction in IgE values; in fact, these remain above normal in patients with EAD despite an optimal clinical response in terms of both the EASI and pruritus reduction [15].

The main limitation of this study is the retrospective methodology. Certainly, larger prospective studies will be needed to confirm the equal efficacy of dupilumab in patients with IAD versus EAD and to further investigate such a topic.

The stratification of AD endotypes could be important for developing personalized medicine approaches that can potentially improve therapeutic outcomes. Indeed, patients with IAD also show significant Th17 and, in some cases, Th22 activation, with increased levels of the relevant cytokines (IL17/IL23 and IL22, respectively). Therefore, such patients could benefit from targeted treatment of these two cytokine axes [9,16].

## Figures and Tables

**Table 1:** Patents’ baseline characteristics.

	IAD (*n* = 49)	EAD (*n* = 311)	*p*-Value
EASI score (mean ± SD)	18.2 ± 11.97	24.04 ± 10.34	<0.01
H&N EASI score (mean ± SD)	2.6 ± 2.09	3.33 ± 2.1	<0.05
DLQI (mean ± SD)	14.13 ± 7.56	14.88 ± 7.07	0.496
POEM (mean ± SD)	19.19 ± 6.56	20.8 ± 6.31	0.094
NRSpp (mean ± SD)	8.63 ± 1.44	8.56 ± 1.75	0.759
NRSsd (mean ± SD)	6.85 ± 2.83	7.07 ± 3.15	0.258
LDH (mean ± SD)	289 ± 125.42	327.12 ± 147.16	0.095
IgE kU/L (mean ± SD)	70.64 ± 60.05	3949.74 ± 5208.77	<0.001
Eosinophils × 10^9^/L (mean ± SD)	0.31 ± 0.39	0.2 ± 9.07	<0.01

IAD: Intrinsic atopic dermatitis; EAD: extrinsic atopic dermatitis; SD: standard deviation; EASI: eczema area severity index; H&N = head and neck; DLQI: dermatology life quality index; POEM: patient-oriented eczema measure; NRSpp: numerical rating scale peak of pruritus; NRSsd: numerical rating scale sleep disturbance.

## Data Availability

The data presented in this study are available on request from the corresponding author. The data are not publicly available due to privacy.
